# Assessment of the Musculoskeletal Axis and Body Composition Parameters in Women with Controlled Thyroid Dysfunction: A Cross-Sectional Study

**DOI:** 10.3390/metabo16090620

**Published:** 2026-08-27

**Authors:** Aleksandra Radecka, Waldemar Pluta, Michał Lubkowski, Anna Lubkowska

**Affiliations:** 1 Department of Functional Diagnostics and Physical Medicine, Pomeranian Medical University, Żołnierska 54, 71-210 Szczecin, Poland; waldemar.pluta@pum.edu.pl; 2 Department of Medical Biology, Pomeranian Medical University, Al. Powstańców Wlkp. 72, 71-210 Szczecin, Poland

**Keywords:** parathyroid hormone, thyroid dysfunction, Hashimoto’s thyroiditis, sarcopenia, bone–muscle axis, body composition, myokines, BDNF, functional assessment

## Abstract

**Background:** The stability of the musculoskeletal axis is crucial for maintaining mobility in women, yet the impact of controlled thyroid dysfunction on this system remains ambiguous. **Objectives**: The aim of this cross-sectional study was to compare the body composition and functional performance of women with Hashimoto’s thyroiditis, women with hypothyroidism, and healthy controls in the context of the role of parathyroid hormone (PTH) as a potential comorbid factor. **Methods**: The study included 70 women divided into three groups: Hashimoto’s thyroiditis (*n* = 27), hypothyroidism (*n* = 15), and control (*n* = 28). Anthropometric parameters, including limb muscle mass (ALM) and bone mineral density (BMD), were assessed using X-ray absorptiometry (DXA). Physical performance was assessed using handgrip strength (HGP) and Timed Up and Go (TUG) tests, supplemented by ELISAs for metabolic and neurotrophic biomarkers (BDNF, irisin, GDF-15, P3NP). **Results:** The results showed no statistically significant differences between groups in muscle mass (ALM: *p* = 0.13; ALMI: *p* = 0.27) or bone density (BMD: *p* = 0.82). Functional performance outcomes were also comparable between groups (HGP: *p* = 0.71; TUG: *p* = 0.143). The levels of the analyzed biomarkers did not differ significantly by thyroid status (BDNF: *p* = 0.91; irisin: *p* = 0.85; GDF-15: *p* = 0.96). Furthermore, correlation analysis revealed that PTH showed a moderate, negative correlation with P3NP in the total population (r = −0.44, *p* = 0.003). In contrast, its association with muscle mass index (ALM) was attenuated after FDR correction. **Conclusions:** These preliminary results indicate no detectable deterioration in musculoskeletal and functional parameters in women with managed thyroid dysfunction. However, due to the small sample size of the hypothyroidism subgroup and low statistical power, these findings cannot establish physiological equivalence and must be interpreted with caution, as they do not confirm a direct protective effect of levothyroxine therapy against muscle mass decline.

## 1. Introduction

The musculoskeletal axis operates as an integrated functional unit whose stability maintains systemic mechanical and metabolic efficiency through bidirectional biochemical crosstalk, safeguarding against age-related musculoskeletal decline [1,2]. While thyroid hormones modulate this axis in both sexes [3], postmenopausal women represent a particularly vulnerable population due to accelerated bone and muscle loss following estrogen decline, combined with a significantly higher prevalence of thyroid dysfunctions [2,4].

The aging process is associated with the progressive degradation of tissues that make up this axis, which can lead to the development of osteosarcopenia—a syndrome characterized by a simultaneous decrease in bone mineral density, muscle mass and strength [5]. The key modulators of this process are thyroid hormones and parathyroid hormone (PTH), whose interrelationships and influence on body composition constitute an important area of endocrine research.

Thyroid dysfunction, including Hashimoto’s disease and hypothyroidism, has a characteristic effect on body weight and its components. As Jonklaas [6] points out, thyroid hormone deficiency promotes fat accumulation and fluid retention, but implementing appropriate replacement therapy and achieving clinical euthyroidism can stabilize the trajectory of these changes. At the same time, a growing body of evidence suggests that parathyroid hormone plays a role beyond the classical regulation of calcium and phosphate metabolism, acting as an active modulator of skeletal muscle metabolism as well as nerve–muscle interactions [7].

PTH (an 84-amino-acid polypeptide; synthesized and secreted by parathyroid glands), calcitriol (1,25-dihydroxyvitamin D; the active form of vitamin D, synthesized by the kidney), and fibroblast growth factor 23 (FGF23; mainly secreted by osteocytes and osteoblasts in bone) are three key hormones modulating calcium and phosphate homeostasis [7,8].

Hashimoto’s disease may indirectly affect parathyroid hormone levels, mainly through vitamin D deficiency, which is common in this condition, and through less common coexisting autoimmune disorders of the parathyroid glands themselves (thyroid and parathyroid gland disorders often go hand in hand due to their proximity and common autoimmune basis). Therefore, fluctuations in PTH levels in people with Hashimoto’s disease may be caused by both unstable calcium and phosphate metabolism and coexisting autoimmune processes, suggesting the importance of monitoring parathyroid hormone levels in this condition [9,10,11].

PTH is involved in calcium, phosphate, and vitamin D homeostasis through its complex actions on the bones, kidneys, and the gastrointestinal tract. However, PTH does not directly act on osteoclasts, as they lack the PTH receptor. Thus, it modulates bone resorption through osteoblasts, which express the PTH receptor protein [12].

The mechanism of PTH’s effect on muscle tissue is based on the expression of the PTHR1 receptor directly in muscle fibers. Chronic elevations in this hormone can activate catabolic pathways, leading to impaired mitochondrial bioenergetics and increased proteolysis, which, in turn, result in reduced muscle strength and mass [13]. PTH also decreases collagen synthesis, which can weaken tendons [14]. These associations are particularly important in the context of the stability of anthropometric parameters and bone mineral density (BMD). George et al. [15] emphasize that lean body mass is a strong predictor of BMD, and that PTH may be a significant confounding factor in this relationship in older individuals.

The endocrine regulation of the musculoskeletal axis extends beyond direct thyroid hormone action. Parathyroid hormone (PTH), synthesized and secreted by the parathyroid glands, has recently emerged as a pivotal modulator of skeletal muscle metabolism through the expression of the PTHR1 receptor directly on myocytes [13]. While physiological pulsatile PTH secretion is essential for bone remodelling, chronic subclinical elevations of PTH—often secondary to subclinical calcium deficiencies or age-related hypovitaminosis D—trigger catabolic signalling pathways in muscle tissue [16,17].

These pathways activate ubiquitin-proteasome proteolysis, impair mitochondrial bioenergetics, and suppress insulin-like growth factor 1 (IGF-1) signalling, thereby accelerating muscle mass loss and functional decline [5,13,16,17]. Furthermore, Hashimoto’s thyroiditis may indirectly perturb this delicate balance. Autoimmune thyroid disorders frequently coexist with subclinical parathyroid dysregulation and vitamin D deficiency, potentially compounding the risk of musculoskeletal deterioration in adult women. Understanding whether PTH acts as an independent factor associated with musculoskeletal decay or merely serves as a non-specific biomarker of chronological aging in euthyroid states is crucial for refining preventative strategies in geriatric endocrinology [16,18].

Modern research indicates that a full understanding of interactions within the musculoskeletal unit requires consideration of biochemical factors such as irisin, BDNF, and GDF-15, which act as signaling molecules in intertissue communication [7]. Irisin, a hormonal myokine, influences bone and energy metabolism and, in many respects, acts analogously to thyroid hormones [19]. In turn, BDNF, although traditionally associated with neuroprotection, also regulates muscle metabolism and synaptic plasticity, making it an important indicator of functional well-being in older people [7]. Additionally, the analysis of markers such as P3NP allows assessment of the dynamics of collagen type III turnover. At the same time, GDF-15 serves as a sensitive indicator of systemic cellular and mitochondrial stress [20].

The use of precise diagnostic methods, such as dual-energy X-ray absorptiometry (DXA), enables reliable assessment of changes in body composition and bone structure due to hormonal fluctuations [21]. An important complement to this assessment is the verification of functional status using standardized tests such as the HGP and the Timed Up and Go (TUG) test. The objective of this cross-sectional study is to evaluate the stability of body composition parameters and circulating biomarkers of the bone–muscle–nerve axis in women with pharmacologically controlled Hashimoto’s thyroiditis and hypothyroidism compared to healthy age-matched controls. We formulated two testable hypotheses a priori: (1) despite clinical and biochemical euthyroidism, women with a history of thyroid dysfunction will exhibit subclinical alterations in tissue remodeling (evidenced by altered P3NP levels) and neuromuscular junction degradation (reflected in CAF concentrations); and (2) parathyroid hormone (PTH) will display a weak-to-moderate negative correlation with appendicular lean mass (ALM) across the total sample as a non-specific marker of biological aging, while exploratory subgroup analyses will be conducted to assess whether the physiological correlation between parathyroid hormone (PTH) and brain-derived neurotrophic factor (BDNF) observed in healthy populations remains statistically detectable in the thyroid pathology subgroups.

## 2. Materials and Methods

The study was conducted at the Department of Functional Diagnostics and Physical Medicine, Pomeranian Medical University, Szczecin, from March to May 2025. The study complied with all applicable institutional and governmental regulations regarding the ethical use of volunteers and with the provisions of the Declaration of Helsinki. The Bioethics Committee of the Pomeranian Medical University approved the study protocol (KB-006/33/2023), and all recruited participants signed written informed consent.

### 2.1. Participants

Participants were recruited via social media and advertisements in outpatient and endocrinology clinics. Inclusion criteria included: (1) age 30–85 years, (2) female gender, (3) documented diagnosis of the specific thyroid condition. Hashimoto’s thyroiditis was diagnosed based on the requirement for levothyroxine therapy combined with elevated anti-TPO antibodies and/or characteristic ultrasound findings, whereas non-autoimmune hypothyroidism was defined as requiring levothyroxine therapy with negative antithyroid antibodies. The control group consisted of healthy women with no history of thyroid disease and negative antithyroid antibodies). Non-autoimmune hypothyroidism was operationally defined as primary hypothyroidism requiring levothyroxine replacement in the presence of negative anti-TPO and anti-Tg antibody titers at diagnosis and at enrollment. This group is etiologically heterogeneous and may include post-surgical, post-radioiodine, drug-induced, and idiopathic (seronegative/atrophic) hypothyroidism; granular data allowing further sub-classification of etiology were not systematically available and are addressed as a limitation (Section 5). Because participants were recruited from multiple outpatient and endocrinology centers, thyroid ultrasound and antibody assays were performed as part of routine clinical care rather than centrally by the study team. Diagnoses of Hashimoto’s thyroiditis were accepted when documentation described sonographic features consistent with chronic autoimmune thyroiditis (diffuse hypoechogenicity and/or heterogeneous parenchymal echotexture, with or without a pseudonodular pattern) together with anti-TPO and/or anti-Tg positivity above the reference threshold of the certifying laboratory (typically anti-TPO > 34 IU/mL for chemiluminescent immunoassay platforms in common clinical use in Poland). Eligibility additionally required documented levothyroxine therapy for a minimum of 6 months at a stable dose; current biochemical euthyroidism (TSH within the certifying laboratory’s reference range, approximately 0.4–4.0 mIU/L, at enrollment) was used as a pragmatic, objective proxy for treatment adequacy and adherence, as detailed dosing history and adherence data (e.g., pill counts) were not centrally recorded 4) providing current TSH, fT3 and fT4 test results TSH, fT3, and fT4 values were obtained from each participant’s most recent clinical laboratory testing (within 3 months prior to enrollment) rather than measured centrally by the study team, and therefore may reflect results from different, though generally comparable, clinically validated immunoassay platforms. We excluded pregnant women and those taking medications or dietary supplements known to affect body composition parameters. A total of 90 individuals responding to social media and clinic advertisements were initially assessed for eligibility. Of these, 20 were excluded for the following reasons: failure to meet inclusion criteria (*n* = 7) or meeting exclusion criteria (pregnancy, use of interfering supplements/medications, or incomplete laboratory results; *n* = 13). Ultimately, the study included 27 women in the Hashimoto’s group, 15 in the hypothyroidism group, and 28 in the control group.

All study participants had normal thyroid function (euthyroidism). Patients in the Hashimoto’s thyroiditis (H) and hypothyroidism groups were under the constant care of an endocrinologist, regularly took prescribed pharmacotherapy, and provided current laboratory test results confirming hormonal stability. In the entire study group, the following parameter values were (median; min–max): TSH 2.15 (0.5–4.0) mIU/L, fT4 1.25 (0.8–1.7) ng/dL, and fT3 3.09 (2.3–3.8) pg/mL. Detailed data for individual subgroups were as follows: in the control group (K) TSH 1.63 (0.5–3.9) mIU/L, fT4 1.39 (1.1–1.7) ng/dL, fT3 3.29 (2.4–3.8) pg/mL; in the group with Hashimoto’s disease (H) TSH 2.25 (0.7–3.9) mIU/L, fT4 1.17 (0.8–1.5) ng/dL, fT3 3.12 (2.4–3.7) pg/mL; and in the group with hypothyroidism TSH 2.87 (1.1–4.0) mIU/L, fT4 1.14 (0.8–1.5) ng/dL and fT3 2.65 (2.3–3.4) pg/mL.

### 2.2. Biochemical Parameters of Venous Blood

Blood was collected by qualified medical personnel, after fasting, in the morning after 15 min resting time, from the flexural vein using Vacutainer tubes (Sarstedt, Nümbrecht, Germany).

Brain-derived neurotrophic factor (BDNF) concentrations were determined using commercially available enzyme-linked immunosorbent assay (ELISA) kits (EIAab, Wuhan, China). The assay range was between 0.156 and 10 ng/mL, intra-assay: ≤6.3% and inter-assay: ≤9.8%.

Growth differentiation factor-15 (GDF-15) concentrations were determined using commercially available enzyme-linked immunosorbent assay (ELISA) kits (EIAab, Wuhan, China). The assay range was between 15.6 and 1000 pg/mL, intra-assay: ≤6.4% and inter-assay: ≤10.3%.

Irisin concentrations were determined using commercially available enzyme-linked immunosorbent assay (ELISA) kits (EIAab, Wuhan, China). The assay range was between 0.78 and 50 ng/mL, intra-assay: ≤5.8% and inter-assay: ≤10.5%.

Procollagen amino-terminal peptide type III (P3NP) concentrations were determined using commercially available enzyme-linked immunosorbent assay (ELISA) kits (EIAab, Wuhan, China). The assay range was between 0.312 and 20 ng/mL, intra-assay: ≤5.5% and inter-assay: ≤8.2%.

Parathyroid hormone (PTH) concentrations were determined using commercially available enzyme-linked immunosorbent assay (ELISA) kits (EIAab, Wuhan, China). The assay range was between 23.4 and 1500 pg/mL, intra-assay: ≤5.6% and inter-assay: ≤9.3%.

C-terminal agrin fragment (CAF) concentrations were determined using commercially available enzyme-linked immunosorbent assay (ELISA) kits (ELK (Wuhan) Biotechnology Co., Ltd., Wuhan, Hubei, China). The assay range was between 31.25 and 2000 pg/mL, intra-assay: ≤8% and inter-assay: ≤10%.

Total serum calcium concentrations were determined using commercially available enzyme-linked immunosorbent assay (ELISA) kits (Cayman Chemical Company, Ann Arbor, MI, USA). The assay range was between 8.9 and 10.4 mg/dL, intra-assay: ≤3% and inter-assay: ≤5.96%.

Due to sample volume restrictions and resource constraints, all ELISA measurements were performed as singletons rather than in duplicates. Furthermore, for samples whose optical density fell outside the standard calibration curve (either above the upper limit or below the lower limit of detection), no additional dilution or concentration protocols were applied. Instead, the final concentrations for these specific out-of-range samples were mathematically extrapolated by the analytical software. Therefore, extreme absolute values reported in this study should be interpreted as estimates.

### 2.3. Body Composition Analysis

Body composition analysis was performed by dual-energy X-ray absorptiometry (DXA) using the Hologic device, QDR 4500W^®^ (Quirugil, Bogota, Colombia) with Hologic APEX software (APEX Version 5.6.0.4, Hologic Inc., Marlborough, MA, USA; https://www.hologic.com) to perform a high-resolution whole-body scan. According to the manufacturer’s recommendations, volunteers were positioned supine with their arms at their sides, their thumbs on their iliac crests, their fingers spread, and their legs rotated inward by 25 degrees. During the test, the patient lay still and breathed normally. The device was calibrated according to the manufacturer’s requirements. To minimize human error, all analyses were performed by a single person. Appendicular lean mass (ALM) was calculated as the sum of the lean soft-tissue mass of both the upper and lower limbs. The bone mineral density (BMD) values reported in this study reflect Total Body BMD. Because site-specific scans (e.g., lumbar spine, femoral neck) were not performed, T-scores and Z-scores were not calculated, and formal diagnostic criteria for osteopenia or osteoporosis were not applied.

### 2.4. Handgrip Strength Test

The study was conducted according to measurement standards recommended by the American Association of Hand Therapists, which include: a standing position during the measurement, the upper limb in a relaxed position, and 90° elbow flexion, without support from the body [22]. Grip strength measurement lasted 2 to 3 s, during which the volunteer was verbally motivated and the test was monitored. Measurements were taken three times for the right and left hands. For the purpose of the final statistical analysis, the average value of the three measurements obtained from the participant’s dominant hand was used. Isometric handgrip strength was assessed using a SAEHAN digital hand dynamometer (DHD-1 Digital Hand Dynamometer, Saehan Corporation, Changwon, Republic of Korea). The DHD-1’s grip strength measurement range is 0 to 90 kg.

### 2.5. Timed Up and Go Test

The test was conducted in accordance with the procedural assumptions described in the literature [23]. A standard chair with a seat height of 45 cm was used. The starting position was sitting with the hands resting on the thighs and the feet resting on the floor in a comfortable position. After the experimenter demonstrated the test, each participant completed the test twice. On the experimenter’s command, the volunteer performed the following task at the fastest pace possible: standing from the chair to a standing position (without using their hands), immediately walking (without the possibility of running) in a straight line for 3 m, turning around and walking back to the chair, and sitting on the chair. Time was measured using an electronic stopwatch (Casio HS-80TW-1EF Stopwatch, Casio, Tokio, Japan), with an accuracy of 0.1 s. The better result from the two trials was included in the analysis.

### 2.6. Statistical Analysis

All statistical analyses were performed using TIBCO STATISTICA software (version 13.3, TIBCO Software Inc., Palo Alto, CA, USA). The study was exploratory in nature, utilizing a convenience sample; thus, a prospective sample-size calculation and a single primary outcome were not defined a priori. Normality of continuous variables was assessed using the Shapiro–Wilk test. The homogeneity of variances was tested using Levene’s test.

For normally distributed variables, results were expressed as mean ± standard deviation (SD) with corresponding 95% confidence intervals (95% CIs) and compared across the three study groups (Hashimoto’s thyroiditis, hypothyroidism, controls) using one-way analysis of variance (ANOVA). F-statistics and *p*-values were reported, with post hoc tests planned if statistical significance had been reached.

For variables with non-normal distributions, or where significant outliers were visually identified via boxplots, non-parametric methods were strictly preferred to avoid the undue influence of extreme values. Extreme physiological outliers (including extrapolated ELISA values) were retained in the dataset to preserve natural sample variance. These results were presented as median values with interquartile ranges (IQRs). Their 95% CIs were estimated using the bootstrap method (based on 10,000 replications, using the percentile method). Intergroup comparisons for these variables were carried out using the Kruskal–Wallis rank-sum test.

Regarding missing data, the final dataset contained no missing values for the included 70 participants, allowing for a complete case analysis.

Associations between parathyroid hormone (PTH) concentrations and metabolic, musculoskeletal, and functional parameters were evaluated using Spearman’s rank correlation analysis. Correlations were examined both in the total study population and within subgroups (controls, Hashimoto’s thyroiditis, hypothyroidism). Results were reported as correlation coefficients (r) with corresponding nominal *p*-values and their Benjamini–Hochberg False Discovery Rate (FDR) adjusted q-values to account for multiple comparisons. For the Benjamini–Hochberg adjustment, families of tests were defined per study group; that is, all correlation tests between PTH and the studied variables within a specific subgroup (e.g., within the Hashimoto group) were treated as a single family. Both nominal *p*-values and FDR-adjusted q-values are reported.

All tests were two-sided, and *p*-values < 0.05 were considered statistically significant. Trends approaching significance (0.05 ≤ *p* < 0.10) were noted as potentially clinically relevant. All statistical analyses were conducted using the STATISTICA software package.

The results of the retrospective power analysis of the analyzed variables are presented in the Supplementary Material (Table S1).

## 3. Results

### 3.1. Study Population Characteristics

The study included 70 participants and included comprehensive assessments of anthropometric, biochemical, and functional parameters. Table 1 presents the detailed characteristics of the study population.

The study population consisted of older adults with a mean age of 58.96 years (95% CI: 56.20–61.72 years). The participants had a mean BMI of 25.83 kg/m^2^ (95% CI: 24.80–26.86 kg/m^2^), which falls within the overweight category according to the WHO classification, although such values are frequently observed and considered within an optimal range in older adult populations. Given this demographic profile, the majority of the participating women were likely postmenopausal, although specific data regarding the exact onset of menopause or prior use of menopausal hormone therapy were not systematically recorded.

The median concentration of brain-derived neurotrophic factor (BDNF) was 1.97 ng/mL (95% CI: 1.44–2.52 ng/mL) with substantial individual variation (range: 0.06–16.00 ng/mL). Growth differentiation factor-15 (GDF-15) had a median of 67.30 pg/mL (95% CI: 58.2–97.4 pg/mL) and wide variability (range: 15.70–431.30 pg/mL). Median irisin concentrations were 1.89 ng/mL (95% CI: 1.81–2.73 ng/mL), with a wide range (0.69–62.25 ng/mL).

Procollagen type III N-terminal propeptide (P3NP) median levels were 5.66 ng/mL (95% CI: 4.95–6.29 ng/mL). Parathyroid hormone (PTH) concentrations were median 68.52 pg/mL (95% CI: 65.13–79.96 pg/mL) within the normal range. Uncorrected total serum calcium levels averaged 7.67 mg/dL (95% CI: 7.55–7.79 mg/dL), which falls below the standard clinical reference range.

C-terminal agrin fragment (CAF) showed a mean of 48.88 pg/mL (95% CI: 42.46–58.1 pg/mL), with extreme variability (range: 26.34–3042.98 pg/mL). ALM averaged 16.51 kg (95% CI: 15.86–17.16 kg). Appendicular lean mass index (ALMI) was 6.12 kg/m^2^ (95% CI: 5.93–6.31 kg/m^2^). Visceral fat mass (VFM) showed substantial variation, with a mean of 590.05 g (95% CI: 526.03–654.07 g). Total body bone mineral density (BMD) averaged 1.04 g/cm^2^ (95% CI: 1.02–1.06 g/cm^2^).Timed Up and Go test (TUG) results had a median of 6.28 s (95% CI: 6.2–6.88 s) with a range of 3.43–12.94 s, indicating good mobility in most participants. HGP had a mean of 25.93 kg (95% CI: 24.90–26.96 kg) and a standard deviation of 4.30 kg.

### 3.2. Non-Normally Distributed Variables in Subgroups

Non-parametric statistical analysis was performed using the Kruskal–Wallis rank-sum test to compare biomarker distributions among three distinct clinical groups: Hashimoto’s thyroiditis patients (*n* = 27), healthy controls (*n* = 28), and hypothyroidism patients (*n* = 15). All analyzed variables demonstrated non-normal distributions, necessitating the use of median values, interquartile ranges (IQR) and 95% confidence intervals (estimated using the bootstrap method) for descriptive statistics.

Procollagen Type III N-Terminal Propeptide (P3NP) showed a trend toward intergroup differences (H = 5.66; *p* = 0.060), suggesting potential clinical relevance, although it did not reach conventional statistical significance. The hypothyroidism group demonstrated notably lower median P3NP concentrations [4.50 ng/mL; IQR: 3.14–5.89] compared to both Hashimoto patients [6.23 ng/mL; IQR: 4.29–39.73] and healthy controls [5.74 ng/mL; IQR: 3.54–14.21]. This pattern suggests potentially altered bone formation activity in hypothyroid patients.

C-terminal agrin fragment (CAF) levels were comparable across groups (*p* = 0.68), with the Hashimoto group showing slightly elevated median concentrations [58.10 pg/mL; IQR: 39.60–68.79] compared to controls [47.72 pg/mL; IQR: 40.06–57.09] and hypothyroidism patients [44.62 pg/mL; IQR: 40.39–65.15].

Brain-derived neurotrophic factor (BDNF) levels were remarkably consistent across all groups (*p* = 0.91), with median concentrations ranging from 1.83 ng/mL in controls to 2.28 ng/mL in Hashimoto patients. Similarly, irisin concentrations showed no significant intergroup variation (*p* = 0.85), with median values clustered between 1.82 and 2.00 ng/mL across all groups.

Growth differentiation factor-15 (GDF-15) demonstrated considerable within-group variability but no significant between-group differences (*p* = 0.96). Notably, the hypothyroidism group exhibited a wide interquartile range [30.93–103.60 pg/mL], suggesting heterogeneous inflammatory responses within this population.

Parathyroid hormone (PTH) concentrations showed variable median patterns but were not statistically significant (*p* = 0.59). Median levels were 66.22 pg/mL in Hashimoto patients, 72.27 pg/mL in controls, and 68.52 pg/mL in hypothyroidism patients. Uncorrected total serum calcium levels were statistically comparable across all groups (*p* = 0.91). However, because these values fall below standard reference limits and albumin correction was not possible, they do not definitively indicate normal mineral balance, but merely a lack of intergroup variation. Detailed medians, 95% CI, H-statistics, and *p*-values of biochemical parameters are provided in Table 2.

### 3.3. Normally Distributed Variables Across Subgroups

In subgroup comparisons of normally distributed variables, one-way ANOVA revealed no significant differences among the Hashimoto group (*n* = 27), the Control group (*n* = 28), and the Hypothyroidism group (*n* = 15) for any assessed parameter (all *p* > 0.05; Table 3). Analysis of age distribution revealed no statistically significant differences among the Hashimoto (60.85 ± 10.51 years), Control (59.46 ± 11.74 years), and Hypothyroidism (54.60 ± 12.72 years) groups (F = 2.73; *p* = 0.255), confirming demographic comparability across the studied cohorts. Body mass index (BMI) means were 26.07 ± 3.30 kg/m^2^, 25.36 ± 3.50 kg/m^2^ and 26.29 ± 6.91 kg/m^2^, respectively (F = 0.29; *p* = 0.75). ALM did not differ significantly (16.57 ± 2.26 kg; 15.85 ± 2.73 kg; 17.62 ± 3.22 kg; F = 2.14; *p* = 0.13). Bone mineral density (BMD) values were similar across groups (1.03 ± 0.10 g/cm^2^; 1.04 ± 0.10 g/cm^2^; 1.04 ± 0.09 g/cm^2^; F = 0.20; *p* = 0.82). Visceral fat mass (VFM) showed no intergroup variation (629.00 ± 256.03 g; 583.61 ± 272.02 g; 534.55 ± 290.22 g; F = 0.60; *p* = 0.56). HGP means were also comparable (25.59 ± 4.57 kg; 25.80 ± 4.48 kg; 26.83 ± 3.61 kg; F = 0.34; *p* = 0.71). Detailed means, standard deviations, F-statistics, and *p*-values are provided in Table 3.

**Table 2:** Biochemical parameters comparison between study groups.

	Hashimoto Group (n = 27)				Control Group (n = 28)				Hypothyroidism Group (n = 15)				H	*p*-Value
Variable	Median [IQR]		95% CI Lower	95% CI Upper	Median [IQR]		95% CI Lower	95% CI Upper	Median [IQR]		95% CI Lower	95% CI Upper		
BDNF [ng/mL]	2.28	0.66–3.39	0.92	2.67	1.83	0.61–3.23	1.05	2.7	2.60	0.61–3.32	0.63	2.83	0.18	0.91
GDF-15 [pg/mL]	56.70	30.93–90.90	42.3	88.2	67.90	36.50–94.10	37.36	80.43	67.30	30.93–103.60	40.42	79.34	0.09	0.96
Irisin [ng/mL]	2.00	1.54–2.60	1.6	2.32	1.94	1.53–2.64	1.62	2.27	1.82	1.52–2.28	1.52	2.16	0.33	0.85
P3NP [ng/mL]	6.23	4.29–39.73	4.98	15.62	5.74	3.54–14.21	4.13	11.79	4.50	3.14–5.89	3.97	5.32	5.66	0.06
PTH [pg/mL]	66.22	50.17–88.10	54.69	77.06	72.27	55.97–109.23	63.41	96.56	68.52	42.65–135.98	48.51	102.32	1.07	0.59
CAF [pg/mL]	58.10	39.60–68.79	42.46	65.65	47.72	40.06–57.09	40.73	52.92	44.62	40.39–65.14	40.92	65.02	0.76	0.68
Calcium [mg/dL]	7.36	7.23–8.24	7.28	8.19	7.38	7.27–8.24	7.33	8.22	7.40	7.31–8.23	7.33	8.2	0.19	0.91

Legend: Data are presented as median [interquartile range] and 95% confidence intervals (estimated using the bootstrap method) for each subgroup. H statistic, Kruskal–Wallis test statistic; *p*-value, two-sided significance level. BDNF—brain-derived neurotrophic factor; GDF-15—growth differentiation factor-15; P3NP—procollagen type III N-terminal propeptide; PTH—parathyroid hormone; CAF, C-terminal agrin fragment.

For non-normal distributions, the Kruskal–Wallis test was used. ALMI showed no significant differences between groups (H = 2.59; *p* = 0.27), with median values of 6.16 kg/m^2^ [IQR: 5.68–6.59] in the Hashimoto group, 5.87 kg/m^2^ [IQR: 5.29–6.49] in the control group, and 6.18 kg/m^2^ [IQR: 5.50–7.16] in the hypothyroidism group. Similarly, the functional assessment using the Timed Up and Go test did not reveal significant intergroup variation (H = 3.89; *p* = 0.143), although the hypothyroidism group showed a slightly lower median time (5.74 s [IQR: 4.73–5.90]) compared to the Hashimoto (6.40 s [IQR: 5.71–6.85]) and control (6.59 s [IQR: 5.70–6.75]) groups. Detailed means, standard deviations, medians, interquartile ranges, F and H statistics, and *p*-values are provided in Table 3.

It is important to note that the retrospective power analysis (detailed in Supplementary Table S1) revealed low statistical power for the main group comparisons (ranging from 5.9% for BMD to 15.9% for ALM), driven primarily by small effect sizes (e.g., *η*^2^ = 0.060 for ALM) and the limited sample size in the hypothyroidism subgroup.

### 3.4. Correlation Analysis

Correlation analysis was performed between PTH concentrations and the analyzed biomarkers, body composition, and functional assessment. The analysis was performed on the entire study population and stratified by subgroups. Detailed coefficients (r) and FDR-adjusted *p*-values are presented in Table 4.

In the total study population, a statistically significant, moderate negative correlation was observed between PTH and the bone turnover marker P3NP (r = −0.44; *p* = 0.003). In contrast, the apparent weak positive association between PTH and BDNF did not reach statistical significance after adjustment for multiple comparisons (r = 0.25; *p* = 0.172). No other significant correlations were observed at the whole-cohort level.

When stratified by subgroups, distinct correlation profiles emerged. Within the healthy control group, PTH displayed a significant, moderate positive correlation with BDNF (r = 0.50; *p* = 0.041) and a strong negative correlation with P3NP (r = −0.57; *p* = 0.030). In the Hashimoto’s thyroiditis subgroup, the inverse relationship between PTH and P3NP remained apparent as a statistical trend approaching significance (r = −0.52; *p* = 0.077), while other correlations did not reach statistical significance in this exploratory subgroup analysis. Remarkably, within the smaller hypothyroidism subgroup, no statistically significant correlations or relevant trends were detected between PTH levels and any of the evaluated parameters (*p* > 0.05 for all comparisons). Detailed results of the correlation analysis are presented in Table 4.

Analysis of anthropometric parameters and fitness tests revealed no statistically significant differences across the three groups. While this lack of significant differences in ALM, BMD, HGP, and TUG test results might suggest a lack of severe musculoskeletal degradation in patients with pharmacologically controlled hypothyroidism and Hashimoto’s thyroiditis, the low statistical power of our analysis precludes conclusions regarding physiological equivalence.

**Table 3:** Comparison of demographic, anthropometric parameters, body composition, and functional assessment between study groups.

Variable	Hashimoto Group (n = 27)				Control Group (n = 28)				Hypothyroidism Group (n = 15)				F/H *	*p*-Value
	Mean/Median *	±SD/IQR *	95% CI Lower	95% CI Upper	Mean/Median *	±SD/IQR *	95% CI Lower	95% CI Upper	Mean/Median *	±SD/IQR *	95% CI Lower	95% CI Upper		
Age [years]	60.85	10.51	56.69	65.01	59.46	11.74	54.91	64.02	54.60	12.72	47.56	61.64	2.73	0.255
BMI [kg/m^2^]	26.07	3.30	24.76	27.38	25.36	3.50	24.00	26.72	26.29	6.91	22.46	30.12	0.29	0.75
ALM [kg]	16.57	2.26	15.68	17.46	15.85	2.73	14.79	16.91	17.62	3.22	15.84	19.4	2.14	0.13
ALMI [kg/m^2^] *	6.16 *	5.68–6.59 *	5.92	6.4	5.87 *	5.29–6.49 *	5.6	6.23	6.18 *	5.50–7.16 *	5.92	6.4	2.59 *	0.27
BMD [g/cm^2^]	1.03	0.10	0.99	1.07	1.04	0.10	1.00	1.08	1.04	0.09	0.99	1.09	0.2	0.82
VFM [g]	629.00	256.03	527.72	730.28	583.61	272.02	478.15	689.07	534.55	290.22	373.83	695.27	0.6	0.56
TUG [seconds] *	6.40 *	5.71–6.85 *	5.8	6.83	6.59 *	5.7–6.75 *	5.84	6.71	5.74 *	4.73–5.9 *	4.7	5.9	3.89 *	0.143
HGP [kg]	25.59	4.57	23.78	27.40	25.80	4.48	24.06	27.54	26.83	3.61	24.83	28.83	0.34	0.71

Legend: Data are presented as mean ± standard deviation (SD) and 95% confidence intervals for normally distributed variables or as median [interquartile range] and 95% confidence intervals (estimated using the bootstrap method) for non-normally distributed variables (*). Comparisons were performed using one-way analysis of variance (ANOVA, F-statistic) or the Kruskal–Wallis test (H statistic) for non-normally distributed variables (*). A *p*-value < 0.05 was considered statistically significant. BMI—body mass index; ALM—appendicular lean mass; BMD—bone mineral density; VFM—visceral fat mass; HGP—handgrip strength; SD—standard deviation; ANOVA—analysis of variance.

**Table 4:** Correlations between PTH concentration and analyzed variables (Benjamini–Hochberg False Discovery Rate (FDR) adjustment).

	Study	Control	Hashimoto	Hypothyroidism
	Group	Group	Group	Group
	(n = 70)	(n = 28)	(n = 27)	(n = 15)
	R	R	R	R
	[*p*-Value]	[*p*-Value]	[*p*-Value]	[*p*-Value]
	(q-Value)	(q-Value)	(q-Value)	(q-Value)
BMI	−0.13 [0.291] (0.472)	−0.20 [0.316] (0.684)	0.20 [0.316] (0.627)	−0.33 [0.108] (0.470)
BDNF	0.25 [0.040] (0.172)	**0.50** [0.006] **(0.041)**	0.24 [0.230] (0.627)	−0.08 [0.993] (0.993)
GDF-15	0.00 [0.994] (0.994)	−0.06 [0.774] (0.984)	0.00 [0.997] (0.997)	0.04 [0.899] (0.993)
P3NP	**−0.44** [<0.001] **(0.003)**	**−0.57** [0.002] **(0.030)**	−0.52 [0.006] (0.077)	−0.11 [0.568] (0.993)
Irisin	0.07 [0.541] (0.645)	0.20 [0.307] (0.684)	0.11 [0.576] (0.804)	−0.05 [0.864] (0.993)
CAF	0.07 [0.546] (0.645)	0.00 [0.993] (0.993)	0.20 [0.337] (0.627)	0.03 [0.904] (0.993)
Calcium	−0.19 [0.115] (0.300)	−0.11 [0.562] (0.968)	−0.35 [0.083] (0.540)	−0.05 [0.850] (0.993)
ALM	−0.26 [0.033] (0.172)	−0.29 [0.131] (0.567)	0.00 [0.993] (0.997)	−0.47 [0.073] (0.470)
ALMI	−0.21 [0.085] (0.276)	−0.21 [0.289] (0.684)	0.11 [0.597] (0.804)	−0.46 [0.088] (0.470)
BMD	−0.10 [0.427] (0.617)	0.03 [0.863] (0.984)	−0.10 [0.619] (0.804)	−0.35 [0.317] (0.993)
VFM	−0.01 [0.914] (0.991)	−0.06 [0.759] (0.984)	−0.05 [0.802] (0.948)	0.08 [0.781] (0.993)
TUG	0.19 [0.166] (0.359)	0.12 [0.596] (0.968)	0.25 [0.265] (0.627)	0.03 [0.937] (0.993)
HGP	−0.17 [0.205] (0.381)	−0.03 [0.908] (0.984)	−0.31 [0.162] (0.627)	0.01 [0.772] (0.993)

Legend: BMI—body mass index; BDNF—brain-derived neurotrophic factor; GDF-15—growth differentiation factor-15; P3NP—Procollagen Type III N-terminal propeptide; PTH—parathyroid hormone; CAF—C-terminal agrin fragment; ALM—appendicular lean mass; ALMI—appendicular lean mass index; BMD—bone mineral density; VFM—visceral fat mass; TUG—Timed Up and Go; HGP—handgrip strength. Significant correlations are marked in bold.

## 4. Discussion

This study examines the correlations between parathyroid hormone (PTH) and selected biomarkers of bone metabolism, neurotrophic factors, and muscle parameters in women with thyroid dysfunction. The results revealed specific correlation patterns, including a negative association between PTH and the bone formation marker P3NP, suggesting a role for PTH in the bone–muscle axis regardless of thyroid status.

### 4.1. Tissue Remodeling and Fibrosis

In the investigated cohort, the amino-terminal propeptide of type III collagen (P3NP) was evaluated as a circulating biomarker reflecting the dynamics of systemic extracellular matrix (ECM) and soft-tissue turnover [24,25]. Although the crude non-parametric comparison suggested a marginal baseline trend toward lower median P3NP levels in the hypothyroid group (4.50 ng/mL) compared to both the Hashimoto’s cohort (6.23 ng/mL) and healthy controls (5.74 ng/mL), this variation did not achieve conventional statistical significance (H = 5.66, *p* = 0.060) and was completely attenuated after False Discovery Rate (FDR) adjustment (*p* = 0.420).

From a physiological standpoint, the absence of a significant deficit in P3NP profiles within our thyroid pathology subgroups aligns with the assumption that maintaining a stable biochemical euthyroid state helps mitigate the classic connective tissue complications of thyroid insufficiency. In uncompensated states, thyroid hormone deficiency is well-documented to suppress dermal and musculoskeletal fibroblast activity, leading to a deceleration of collagen synthesis and delayed ECM remodeling [26,27]. Such hypometabolic states typically precipitate the clinical accumulation of glycosaminoglycans, contributing to arthralgia and objective muscle stiffness [28,29]. By demonstrating P3NP profiles indistinguishable from healthy age-matched controls, our cross-sectional data are compatible with the possibility that adequate pharmacological compensation via levothyroxine (L-T4) therapy is associated with preserved peripheral collagen type III dynamics; however, in the absence of pretreatment or untreated comparator data, a protective or causal effect of L-T4 on soft-tissue turnover cannot be established from the present design.

Interestingly, the correlation analysis between PTH and P3NP revealed distinct patterns across the subgroups after accounting for multiple comparisons (Table 4). A significant, robust negative correlation was sustained within the healthy control cohort (*r* = −0.57, *p* = 0.030), suggesting a tightly regulated physiological coupling of calcium-regulating hormones and soft-tissue matrix turnover in aging women. In the Hashimoto’s disease group, this inverse relationship was slightly attenuated, presenting as a statistical trend approaching significance (*r* = −0.52, *p* = 0.077). In the exploratory analysis of the hypothyroidism subgroup, this correlation was not statistically significant (r = −0.11). While this difference in significance across subgroups is notable, without formal interaction testing in larger cohorts, it cannot be definitively interpreted as a physiological dissociation or metabolic uncoupling. The markedly reduced sample size within the hypothyroid subgroup (n = 15) severely restricts statistical power to detect weak-to-moderate associations; this apparent uncoupling is therefore more likely a statistical artifact rather than a definitive pathological phenomenon [16,25,30].

### 4.2. Neuromuscular Remodeling Marker

In our cohort, CAF concentrations were comparable across all groups (*p* = 0.68), with only slightly higher median values (58.10 pg/mL) observed in the group with Hashimoto’s thyroiditis compared to the control (47.72 pg/mL) and the hypothyroid group (44.62 pg/mL). The lack of significant differences in CAF concentrations between groups indicates no statistically detectable evidence of neuromuscular junction degradation in this sample. Together with normal HGP and TUG results, this observation is consistent with, but does not by itself confirm, preserved neuromuscular integrity in women with controlled Hashimoto’s thyroiditis and hypothyroidism. To the best of our knowledge, this is one of the first studies to evaluate this parameter in the context of controlled thyroid dysfunction in women, which makes direct comparisons with the literature difficult.

### 4.3. Neurotrophic and Metabolic Markers

Brain-derived neurotrophic factor (BDNF) did not show significant differences between the studied groups (*p* = 0.91), suggesting that systemic neurotrophic potential is maintained in women with stable, managed thyroid disease. This finding is consistent with prior reports linking adequately treated hypothyroidism to preserved circulating BDNF concentrations [27,28]. However, because our design lacks pretreatment or untreated comparator data, these cross-sectional observations cannot establish that levothyroxine therapy itself preserves neurotrophin signaling or musculoskeletal cross-talk in this population; they are compatible with, but do not confirm, this hypothesis [31,32,33,34].

In the case of irisin, the lack of significant differences in concentrations between the study groups (*p* = 0.85) suggests that in women with pharmacologically controlled thyroid function, there are no pathological changes in the secretion of this myokine. This result is consistent with meta-analyses, which report that achieving euthyroidism with levothyroxine treatment normalizes irisin concentrations, which then return to levels observed in healthy individuals [35]. Prior studies attribute this pattern to a substitutive effect of L-T4 on irisin secretion [36] our cross-sectional data cannot confirm this mechanism directly but are not inconsistent with it. The complexity of the thyroid–irisin relationship is underscored by the fact that, in pathological conditions, the phenomenon of so-called “irisin resistance” may occur, in which the initial compensatory secretion of myokine is exhausted as organ damage progresses [19]. In contrast to our results, studies in patients with newly diagnosed, untreated hypothyroidism showed significantly lower irisin levels, which the authors attributed to progressive skeletal muscle degradation [19]. Moreover, the literature indicates that in healthy individuals, irisin levels are negatively correlated with age due to the natural loss of muscle mass; however, in states of thyroid dysfunction, metabolic factors may mask these physiological aging processes [19]. Given that muscles and bones are inextricably linked through complex biochemical signaling, an important element of our analysis was also to investigate the relationship between irisin and parathyroid hormone (PTH) [7]. However, in our cohort, correlation analysis did not reveal a significant association between these parameters (*p* > 0.05), suggesting a regulatory balance within the musculoskeletal unit in treated patients. These results contrast with reports indicating an interaction in which parathyroid hormone acts as a direct negative regulator of irisin secretion, particularly in clinical hyperparathyroidism [37].

The last parameter analyzed was GDF-15, which did not show significant intergroup differences in the studied cohort (*p* = 0.96), suggesting that systemic cellular stress remains stable in women with pharmacologically controlled thyroid dysfunction. This result is consistent with clinical observations, which show a significant increase in GDF-15 concentration mainly in patients with overt hyperthyroidism. In contrast, in hypothyroidism, these levels do not differ significantly from the norm [38]. It is worth noting that GDF-15 is considered a sensitive cytokine whose serum concentration correlates with thyroid function, showing a positive relationship with TSH level and a negative relationship with free thyroxine [20]. The stable median values in our groups (56.70–67.90 pg/mL) may therefore indicate effective metabolic control in the patients. Interestingly, the lowest values observed in the Hashimoto’s disease group (56.70 pg/mL) are consistent with reports suggesting that the presence of antithyroid antibodies (TPO-Ab) may paradoxically lower this marker [20]. As a pleiotropic stress-response cytokine, GDF-15 plays key roles in regulating appetite, cell survival, and inflammatory processes, making it an important indicator of metabolic well-being in endocrinopathies [39]. Although GDF-15 is primarily used for differential diagnosis of thyroid hyperplasia, its stability in our elderly women population supports the maintenance of cellular homeostasis [40]. The lack of correlation between GDF-15 and parathyroid hormone levels (r ≈ 0.00) in the studied cohort further indicates that mitochondrial stress mechanisms are not directly linked to the axial regulation of PTH to the same extent as in neuromuscular regeneration (BDNF) or connective tissue remodeling (P3NP).

### 4.4. Body Composition Assessment and PTH

In our study, analysis of body composition parameters (Table 3) did not reveal statistically significant differences between the study groups in BMI (*p* = 0.75), ALM (*p* = 0.13), or BMD (*p* = 0.82). No statistically detectable differences in BMI, ALM, or BMD were observed between groups in this sample, which is compatible with, but does not establish, preserved musculoskeletal homeostasis in women with controlled Hashimoto’s thyroiditis and hypothyroidism. These observations appear to contrast with the data of Malczyk et al. [41], who reported significantly higher body mass and BMI values in women with HT. However, in the cited study, these differences were mainly due to a higher percentage of adipose tissue (AFM), with no change in fat-free mass (FFM).

A key reference point for interpreting these discrepancies is the recent review by Jonklaas [6], which indicates that hypothyroidism is usually associated with only a “mild to moderate” increase in body weight, and that a significant portion of this increase is due to fluid retention (myxedema) and glycosaminoglycan accumulation, not solely to fat gain [6]. Jonklaas also emphasizes that after initiating levothyroxine treatment and achieving euthyroidism, the fluid retention component is reduced, stabilizing the patients’ body composition [6].

The fact that no differences in ALM or BMI were observed in our group of older adults is consistent with the findings of Garin et al. [42] and the meta-analysis by Kretli-Souza et al. [43], which suggest no clinically significant effect of subclinical or pharmacologically controlled hypothyroidism on body composition in this age group. This is also confirmed by the study by Adamska et al. [44], which indicates an identical body composition profile in euthyroid women with HT and healthy volunteers. Chen et al. [19], in turn, suggest that the risk of sarcopenia in this population is more strongly correlated with free triiodothyronine (fT3) levels than with the autoimmune process itself. The lack of functional differences in our tests (HGP *p* = 0.71; TUG *p* = 0.143) supports Jonklaas’s [6] thesis that, with appropriate treatment, the trajectory of body composition changes becomes level.

The role of parathyroid hormone (PTH) as a potential structural modulator of the musculoskeletal axis has garnered significant research interest, given that chronic hyperparathyroidism is well-documented to trigger catabolic pathways and muscle proteolysis via the direct expression of the PTHR1 receptor on myocytes [7,13]. In our cohort, while the unadjusted baseline analysis suggested a tentative inverse relationship between PTH and ALM across the total sample (r = −0.26), this association did not withstand False Discovery Rate (FDR) correction (*p* = 0.172), remaining statistically non-significant. Furthermore, no significant correlations between PTH and lean mass indices were observed within the clinical subgroups (Table 4).

This attenuation after FDR correction indicates that, in this sample, PTH concentrations were not statistically associated with ALM once multiple testing was accounted for. This cross-sectional finding does not permit inference about whether PTH drives or fails to drive muscle degradation over time. These observations closely align with the data of Valiña-Tóth et al. [45], demonstrating that lean body mass parameters can remain independent of circulating PTH levels when systemic mineral and calcium homeostasis is successfully maintained.

Consequently, when evaluated alongside the stable anthropometric and functional profiles detailed in Table 3, and in light of the clinical consensus outlined by Jonklaas [6] and the meta-analysis by Kretli-Souza et al. [43], our findings are hypothesis-generating regarding a possible association between long-term levothyroxine therapy and stable bone–muscle unit parameters; a primary, independent catabolic role of PTH in this population is not supported by the present cross-sectional correlations, although this cannot be equated with the absence of such a role over time.

### 4.5. Functional Assessment in the Context of Thyroid Dysfunction

Maintaining stability in body composition parameters, particularly appendicular muscle mass (ALM) and the ALMI, is directly reflected in the functional assessment results of the studied patients. In our cohort, no statistically significant differences were found in HGP (*p* = 0.71) or mobility assessed by the Timed Up and Go test (*p* = 0.143) between the Hashimoto’s thyroiditis, hypothyroidism, and control groups (Table 3). The absence of functional deficits on these basic tests suggests no overt functional disability in the studied women. However, given the study’s limited statistical power, we cannot definitively conclude that complete musculoskeletal homeostasis is maintained.

Our observations are directly supported by the results of a pilot study by Gallo et al. [46], conducted among patients with newly diagnosed hypothyroidism (both overt and subclinical). These authors demonstrated that while patients with hypothyroidism exhibited significantly more neuromuscular symptoms and higher creatine kinase levels, differences in HGP and the chair rise test did not reach statistical significance compared to the control group [46]. This result is consistent with our data and suggests that static muscle strength (as measured by HGP) may remain relatively preserved even in the early stages of thyroid dysfunction. Furthermore, Gallo et al. [46] reported normalization of functional parameters following levothyroxine therapy; our findings in a stably treated cohort are consistent with, although do not directly confirm, this observation.

It should be noted, however, that Gallo et al. [46] indicated the 6 min walk test (6MWT) as the most valuable and sensitive tool for detecting subclinical abnormalities in physical performance, which were significantly reduced in their patients [46]. Similar conclusions are drawn from the work of Tanriverdi et al. [47], where women with newly diagnosed subclinical hypothyroidism had poorer 6 MWT results and reduced quadriceps strength [47]. The lack of differences in our study using the TUG and HGP tests may therefore be because these tests assess short-term, low-intensity exercise, which—as also suggested by Lankhaar et al. [30]—may not sufficiently burden the metabolic and cardiorespiratory systems to reveal subtle deficits typical of patients with hypothyroidism.

The normal functional results in our group are also consistent with the reports of Chen et al. [19], according to which it is the level of free triiodothyronine (fT3), not autoimmune status (anti-TPO), that determines the risk of sarcopenia and disability in euthyroid patients with Hashimoto’s thyroiditis [19]. The lack of correlation between PTH and functional test results in our study is consistent with the absence of a detectable adverse association between PTH and motor function in this sample with preserved calcium–phosphate homeostasis and the absence of chronic hyperparathyroidism, as described by Romagnoli and Brandi [13] and Nguyen et al. [7]. These cross-sectional findings should be interpreted cautiously and do not establish that replacement therapy caused or preserved functional capacity.

The lack of significant differences between the HGP and TUG tests in our cohort may be due to their nature as screening tools designed to detect overt functional disability. In able-bodied individuals living in the community, these tests may exhibit a so-called ceiling effect, limiting their ability to differentiate subtle metabolic deficits. Nevertheless, the stability of these results in patients with HT and hypothyroidism is an important clinical indicator, suggesting that the current metabolic status of the women studied allows them to maintain motor skills at the level of their peers without thyroid dysfunction.

## 5. Limitations

While providing valuable insights into the musculoskeletal status of euthyroid women, several methodological limitations must be addressed when interpreting our findings. First, due to the cross-sectional nature of the study, the results reflect the patients’ clinical status at a single point in time, which prevents the establishment of direct causal relationships between parathyroid hormone dynamics and muscle parameters, as well as a comprehensive analysis of long-term body composition dynamics. Second, the sample size of the hypothyroid subgroup (*n* = 15) was relatively small, potentially limiting our statistical power to detect highly subtle intergroup differences that might emerge in larger cohorts. We did not perform a prospective a priori sample-size calculation or designate a single primary outcome, which significantly limits our statistical power to definitively confirm the absence of subtle physiological differences between groups. Consequently, our findings must be treated as hypothesis-generating rather than definitive.

From a clinical and epidemiological perspective, the inability to perform multivariate adjusted analyses represents a major constraint. Our unadjusted models did not systematically account for several critical confounding variables. Specifically, we lacked data on renal function, dietary calcium intake, serum phosphate, serum magnesium, specific relevant comorbidities, and concurrent medication use, all of which dynamically influence mineral homeostasis and muscle metabolism. Furthermore, we lacked serum 25-hydroxyvitamin D (25(OH)D) and serum albumin measurements; given that hypovitaminosis D directly modulates PTH secretion and skeletal muscle health, its omission restricts our ability to definitively separate the direct effects of PTH from secondary hyperparathyroidism. Additionally, the lack of detailed historical data regarding the exact dose and duration of levothyroxine replacement therapy, as well as antibody titers prior to enrollment, restricts our ability to analyze the impact of chronic exposure to thyroid dysfunction. Furthermore, because thyroid ultrasound, antithyroid antibody titers, and TSH/fT3/fT4 concentrations were obtained from participants’ own clinical records rather than measured centrally on a single standardized assay platform, some degree of inter-laboratory and inter-platform variability cannot be excluded. Similarly, the non-autoimmune hypothyroidism subgroup was etiologically heterogeneous, and we were unable to systematically differentiate post-surgical, post-radioiodine, drug-induced, and idiopathic seronegative causes.

Furthermore, regarding functional and demographic criteria, the inclusion of a wide age range (30–85 years) without systematic assessment of menopausal status introduces a significant confounding factor. The menopausal transition and subsequent estrogen deficiency are primary drivers of accelerated bone loss. Since we did not stratify our cohort by years since menopause or account for potential past menopausal hormone therapy, we cannot fully isolate the influence of thyroid status and parathyroid hormone on bone mineral density (BMD) from the overarching effects of physiological reproductive aging. Furthermore, a major limitation of our study is the lack of standardized evaluation of physical activity levels (e.g., using validated questionnaires to objectively classify participants as sedentary or actively exercising). Because lifestyle behaviors, particularly regular resistance or aerobic training, profoundly impact muscle mass (ALM), HGP, and functional performance (TUG), the absence of this stratification restricts our ability to completely isolate the effects of thyroid dysfunction from these crucial biomechanical confounders. Although our cohort consisted of community-dwelling women without professional athletic backgrounds, future studies must strictly incorporate comprehensive physical activity tracking to accurately interpret musculoskeletal outcomes in this population. Although chronological age was adjusted for in our partial correlation models, future studies must incorporate comprehensive physical activity questionnaires and utilize age- and menopause-stratified cohorts to fully elucidate these relationships. Additionally, the functional tests employed (HGP, TUG), while standard and reliable clinical tools, focus primarily on basic motor skills and static strength. Consequently, they may not fully capture alterations in functional reserve or aerobic capacity, which are more likely to be revealed under higher exercise loads. Future prospective longitudinal studies with larger cohorts and complete calcitropic and functional profiles are warranted to further validate these findings.

Furthermore, a notable laboratory limitation is that the ELISA biomarker assays were performed as single measurements rather than duplicates, and values falling outside the manufacturer’s validated assay ranges were software-extrapolated rather than diluted and re-assayed. This restricts the quantitative precision of the most extreme biomarker concentrations and warrants cautious interpretation of the absolute values.

Finally, our study design evaluated exclusively patients who had achieved biochemical euthyroidism through levothyroxine replacement therapy. While the inclusion of a control group comprising patients with uncompensated, untreated hypothyroidism would have provided a valuable comparative baseline for musculoskeletal degradation, such a design was precluded by ethical constraints. Withholding standard-of-care hormone replacement in women with diagnosed thyroid failure is ethically impermissible. Consequently, our findings are strictly applicable to the clinical scenario of pharmacologically managed thyroid dysfunction, and we cannot directly quantify the baseline tissue degradation that would have occurred without intervention.

## 6. Conclusions

This study observed no statistically significant differences in body composition (ALM, BMD), functional capacity (HGP, TUG), or neurotrophic/metabolic profiles (BDNF, irisin, GDF-15) between healthy controls and women with pharmacologically controlled Hashimoto’s thyroiditis and hypothyroidism. No statistically detectable differences in musculoskeletal or functional parameters were observed between groups in this sample; however, the study was not powered to confirm physiological equivalence, and a mitigating effect of euthyroidism cannot be inferred from this cross-sectional design. The observed lack of statistical significance should not be interpreted as absolute proof of preserved homeostasis. Further appropriately powered, longitudinal studies with prespecified equivalence margins are required to definitively determine the protective effects of levothyroxine on the bone–muscle axis.

Despite clinical stability, exploratory analyses suggest potential variations in the relationship between PTH and BDNF in patients with hypothyroidism; however, further formal interaction testing in larger cohorts is required to confirm these preliminary observations. In the analyzed cohort, parathyroid hormone serves only as a general marker of aging processes, not as a specific factor correlated with muscle changes directly related to thyroid pathophysiology. In this sample, controlled thyroid dysfunction was not associated with statistically detectable musculoskeletal impairment; longitudinal studies are needed to determine whether this reflects a true absence of risk or a limitation of statistical power.

## 7. Clinical Implications

While these findings suggest that overt musculoskeletal degradation may be mitigated by achieving euthyroidism, the study was not powered to confirm physiological equivalence. These findings should be regarded as hypothesis-generating rather than as evidence of a direct musculoskeletal benefit of levothyroxine therapy, given the cross-sectional design and the absence of pretreatment or untreated comparator data. Within this sample, no statistically detectable differences in body composition, functional capacity, or the assessed biomarkers were observed between women with pharmacologically managed Hashimoto’s thyroiditis or hypothyroidism and healthy controls. This observation may be compatible with adequate musculoskeletal status in stably treated patients, but it does not establish that levothyroxine therapy causes, protects, or normalizes these outcomes. The trend toward lower P3NP concentrations in the hypothyroidism subgroup and the subgroup-dependent PTH–BDNF correlation pattern warrant further investigation in adequately powered, longitudinal studies before any clinical recommendation regarding supplementary functional testing or physical activity interventions can be made. Given that other studies using more demanding functional tests (e.g., 6 min walk test) have detected subclinical deficits not captured by HGP or TUG [42,43], our data should not be interpreted as indicating that advanced functional assessment is unnecessary in this population; this question remains open and requires dedicated study.

## 8. Declaration of Generative Artificial Intelligence and AI-Assisted Technologies in the Abstract Creation Process

During the preparation of this manuscript, the authors used the Gemini 3.1 Pro language model (Google LLC; accessed 3 August 2026) to generate a preliminary graphical abstract. The AI was prompted by the abstract text. After using this AI tool, the authors subjected the resulting image to extensive manual editing using digital illustration software to standardize fonts, improve the generated text, and ensure a precise and clear visual representation of the abstract. The authors reviewed the final visual content and are fully and solely responsible for the scientific accuracy and integrity of the published graphics.

## Figures and Tables

**Table 1:** Characteristics of the study population (N = 70).

Parameter	Mean/Median *	95% CI Lower	95% CI Upper	SD/Min-Max *
BMI [kg/m^2^]	25.83	24.80	26.86	4.33
Age [years]	58.96	56.20	61.72	11.57
BDNF [ng/mL] *	1.97 *	1.44	2.52	0.06–16 *
GDF-15 [pg/mL] *	67.30 *	58.2	97.4	15.70–431.3 *
Irisin [ng/mL] *	1.89 *	1.81	2.73	0.69–62.25 *
P3NP [ng/mL] *	5.66 *	4.95	6.29	1.23–106.16 *
PTH [pg/mL] *	68.52 *	65.13	79.96	17.32–367.45 *
CAF [pg/mL] *	48.88 *	42.46	58.1	26.34–3042.98 *
Calcium [mg/dL]	7.67	7.55	7.79	0.52
ALM [kg]	16.51	15.86	17.16	2.72
ALMI [kg/m^2^]	6.12	5.93	6.31	0.80
BMD [g/cm^2^]	1.04	1.02	1.06	0.09
VFM [g]	590.05	526.03	654.07	268.51
TUG [seconds] *	6.28 *	6.2	6.88	3.43–12.94 *
HGP [kg]	25.93	24.90	26.96	4.30

Legend: For normally distributed parameters, results are presented as mean, standard deviation (SD), and 95% confidence intervals (95% CIs). For non-normally distributed parameters (*), values are presented as medians, minimum–maximum ranges, and 95% confidence intervals estimated using the bootstrap method. BMI—body mass index; BDNF—brain-derived neurotrophic factor; GDF-15—growth differentiation factor-15; P3NP—procollagen type III N-terminal propeptide; PTH—parathyroid hormone; CAF—agrin C-terminal fragment; ALM—lean limb mass; ALMI—lean limb mass index; BMD—bone mineral density; VFM—visceral fat mass; TUG—Timed Up and; HGP—handgrip strength.

## Data Availability

The data presented in this study are available on request from the corresponding author due to privacy and ethical restrictions.

## Supplementary Materials

The following supporting information can be downloaded at: https://www.mdpi.com/article/10.3390/metabo16090620/s1, Table S1: Retrospective power analysis.
