# Supplementary material for: Assessment of the Musculoskeletal Axis and Body Composition Parameters in Women with Controlled Thyroid Dysfunction: A Cross-Sectional Study
Source: Metabolites. 2026 Aug 27;16(9):620. doi: 10.3390/metabo16090620 (PMC13609725; doi:10.3390/metabo16090620)
Supplement: Supplementary file 1 [file metabolites-16-00620-s001.zip › metabolites-4466524-supplementary.pdf]

Table S1. Retrospective power analysis

variable	Effect size	Power of the test (1-β)
weight	f Cohena = 0.120<br>(η^2 = 0.014)	**7.3%**
height	f Cohena = 0.136<br>(η^2 = 0.018)	**7.9%**
BMI	f Cohena = 0.092<br>(η^2 = 0.008)	**6.3%**
age	f Cohena = 0.210<br>(η^2 = 0.042)	**12.3%**
BDNF [ng/mL]	f Cohena = 0.103<br>(η^2 = 0.010)	**6.6%**
GDF-15 [pg/mL]	f Cohena = 0.018<br>(η^2 = 0.000)	**5.1%**
Irisin [ng/mL]	f Cohena = 0.115<br>(η^2 = 0.013)	**7.1%**
P3NP [ng/mL]	f Cohena = 0.286<br>(η^2 = 0.075)	**18.7%**
PTH [pg/ml]	f Cohena = 0.012<br>(η^2 = 0.000)	**5.0%**
CAF [pg/nl]	f Cohena = 0.031<br>(η^2 = 0.001)	**5.1%**
Calcium [mg/dl]	f Cohena = 0.066<br>(η^2 = 0.004)	**5.7%**
ALM [kg]	f Cohena = 0.253<br>(η^2 = 0.060)	**15.9%**
ALMI [kg/m2]	f Cohena = 0.247<br>(η^2 = 0.058)	**15.4%**
BMD	f Cohena = 0.078<br>(η^2 = 0.006)	**5.9%**
VFM	f Cohena = 0.134<br>(η^2 = 0.018)	**7.8%**
TUG	f Cohena = 0.251<br>(η^2 = 0.059)	**12.9%**
HGP	f Cohena = 0.112<br>(η^2 = 0.012)	**6.5%**
